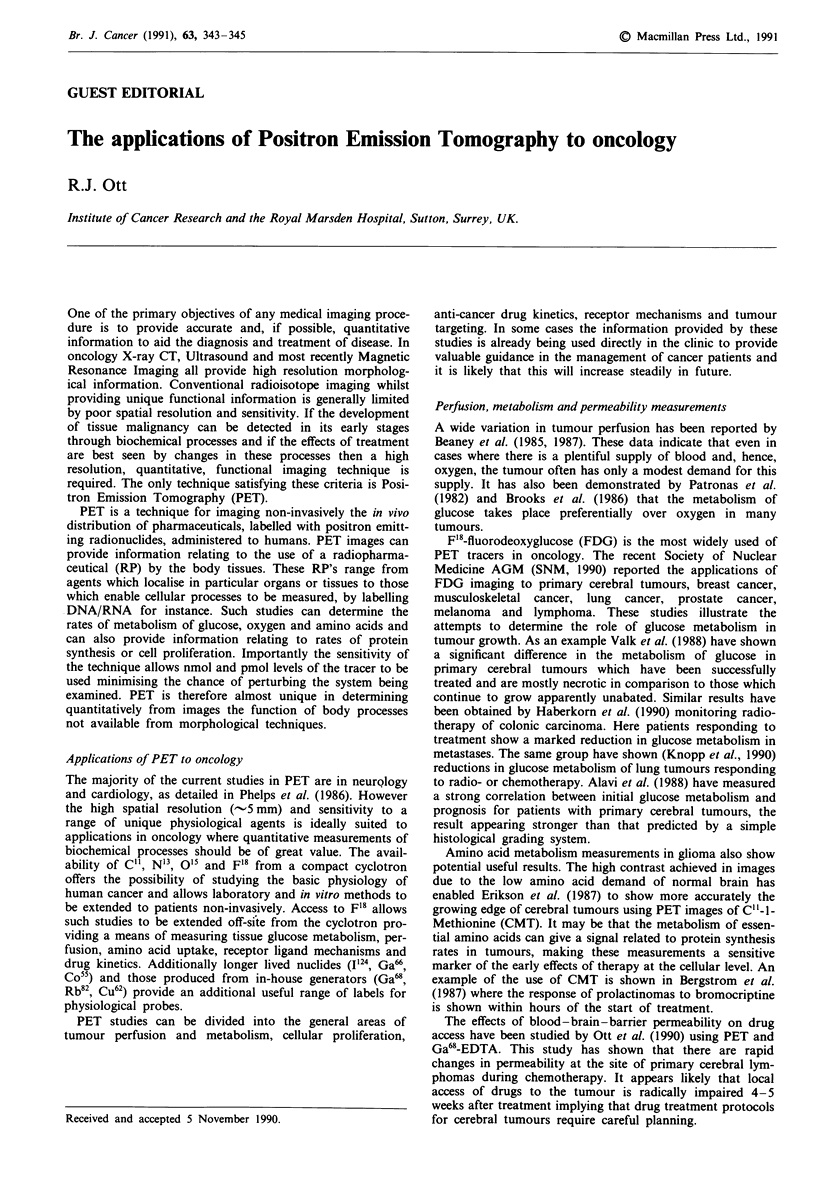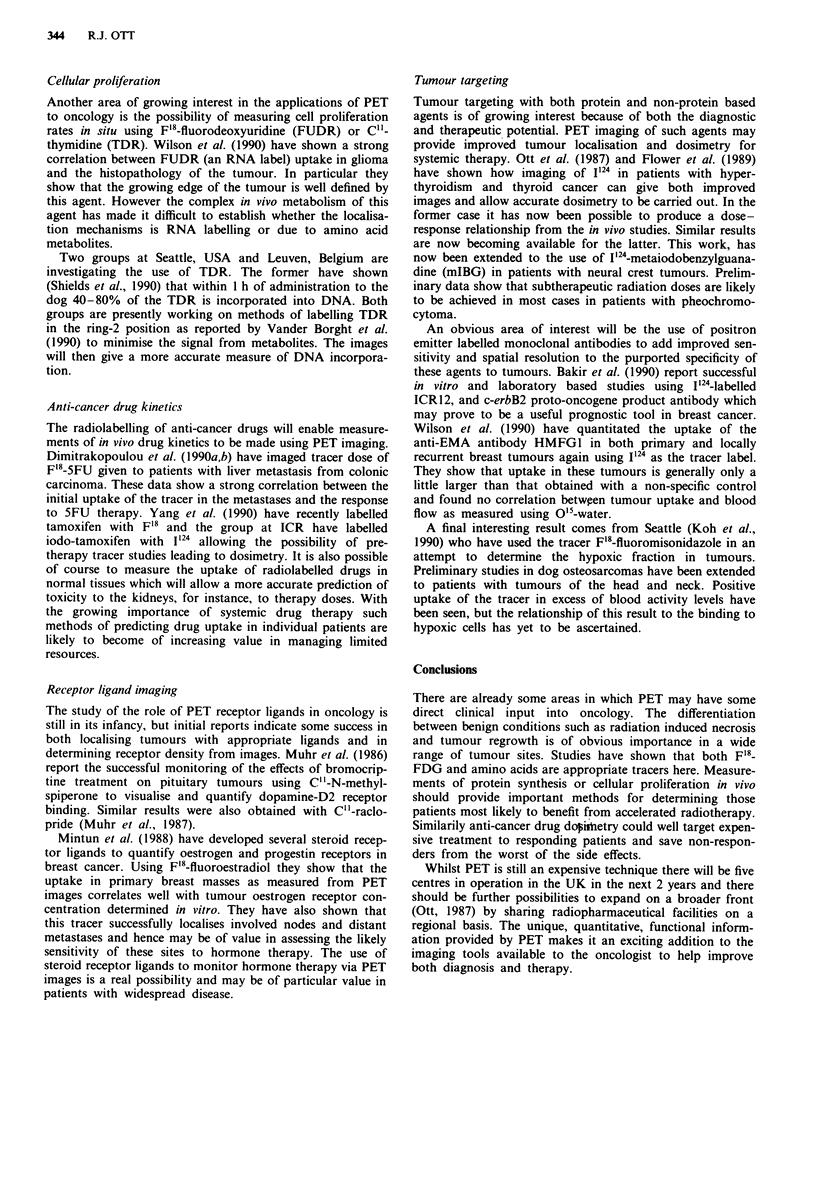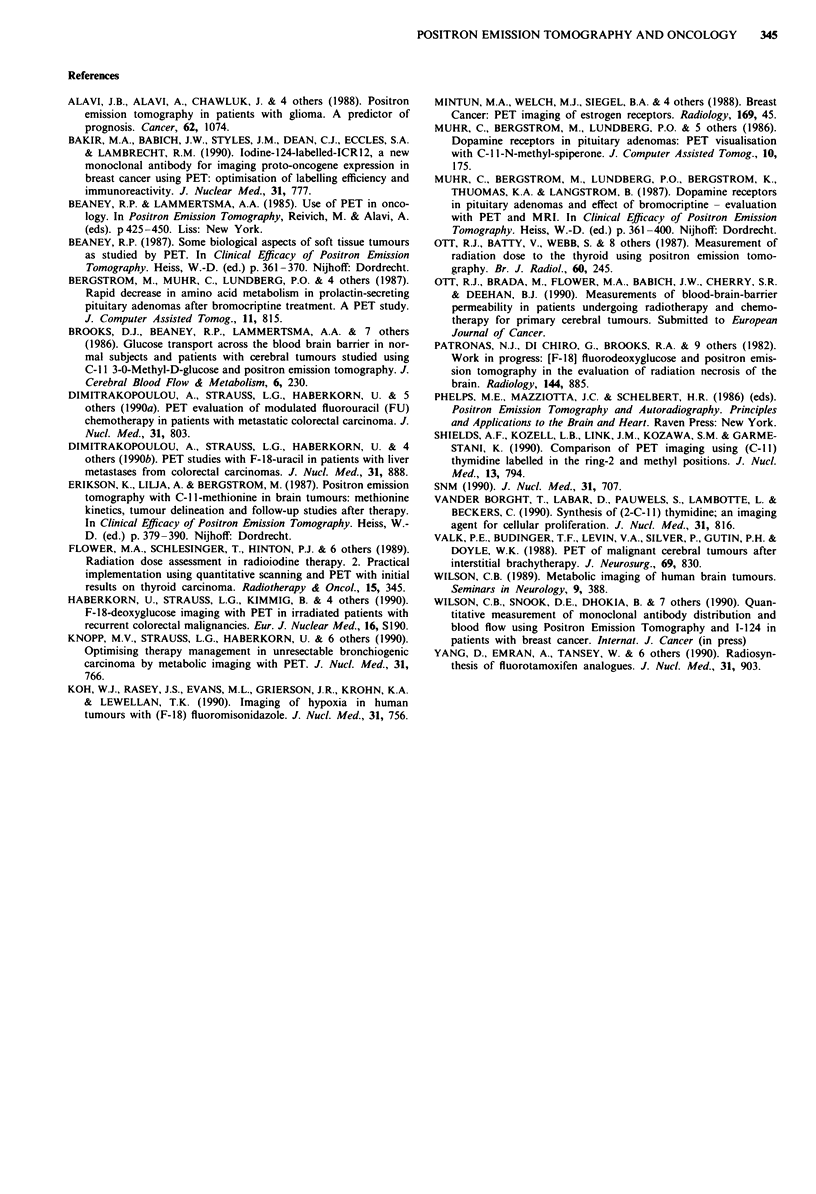# The applications of positron emission tomography to oncology.

**DOI:** 10.1038/bjc.1991.81

**Published:** 1991-03

**Authors:** R. J. Ott


Br. J. Cancer (1991), 63, 343-345 © Macmillan Press Ltd., 1991

GUEST EDITORIAL

### The applications of Positron Emission Tomography to oncology

R.J. Ott

*Institute of Cancer Research and the Royal Marsden Hospital, Sutton, Surrey, UK.*

One of the primary objectives of any medical imaging procedure is to provide accurate and, if possible, quantitative information to aid the diagnosis and treatment of disease. In oncology X-ray CT, Ultrasound and most recently Magnetic Resonance Imaging all provide high resolution morphological information. Conventional radioisotope imaging whilst providing unique functional information is generally limited by poor spatial resolution and sensitivity. If the development of tissue malignancy can be detected in its early stages through biochemical processes and if the effects of treatment are best seen by changes in these processes then a high resolution, quantitative, functional imaging technique is required. The only technique satisfying these criteria is Positron Emission Tomography (PET).

PET is a technique for imaging non-invasively the *in vivo* distribution of pharmaceuticals, labelled with positron emitting radionuclides, administered to humans. PET images can provide information relating to the use of a radiopharmaceutical (RP) by the body tissues. These RP's range from agents which localise in particular organs or tissues to those which enable cellular processes to be measured, by labelling DNA/RNA for instance. Such studies can determine the rates of metabolism of glucose, oxygen and amino acids and can also provide information relating to rates of protein synthesis or cell proliferation. Importantly the sensitivity of the technique allows nmol and pmol levels of the tracer to be used minimising the chance of perturbing the system being examined. PET is therefore almost unique in determining quantitatively from images the function of body processes not available from morphological techniques.

### *Applications of PET to oncology*

The majority of the current studies in PET are in neurology and cardiology, as detailed in Phelps *et al.* (1986). However the high spatial resolution (~5 mm) and sensitivity to a range of unique physiological agents is ideally suited to applications in oncology where quantitative measurements of biochemical processes should be of great value. The availability of $C^{11}$, $N^{13}$, $O^{15}$ and $F^{18}$ from a compact cyclotron offers the possibility of studying the basic physiology of human cancer and allows laboratory and *in vitro* methods to be extended to patients non-invasively. Access to $F^{18}$ allows such studies to be extended off-site from the cyclotron providing a means of measuring tissue glucose metabolism, perfusion, amino acid uptake, receptor ligand mechanisms and drug kinetics. Additionally longer lived nuclides ($I^{124}$, $Ga^{66}$, $Co^{55}$) and those produced from in-house generators ($Ga^{68}$, $Rb^{82}$, $Cu^{62}$) provide an additional useful range of labels for physiological probes.

PET studies can be divided into the general areas of tumour perfusion and metabolism, cellular proliferation, anti-cancer drug kinetics, receptor mechanisms and tumour targeting. In some cases the information provided by these studies is already being used directly in the clinic to provide valuable guidance in the management of cancer patients and it is likely that this will increase steadily in future.

### *Perfusion, metabolism and permeability measurements*

A wide variation in tumour perfusion has been reported by Beaney *et al.* (1985, 1987). These data indicate that even in cases where there is a plentiful supply of blood and, hence, oxygen, the tumour often has only a modest demand for this supply. It has also been demonstrated by Patronas *et al.* (1982) and Brooks *et al.* (1986) that the metabolism of glucose takes place preferentially over oxygen in many tumours.

$F^{18}$-fluorodeoxyglucose (FDG) is the most widely used of PET tracers in oncology. The recent Society of Nuclear Medicine AGM (SNM, 1990) reported the applications of FDG imaging to primary cerebral tumours, breast cancer, musculoskeletal cancer, lung cancer, prostate cancer, melanoma and lymphoma. These studies illustrate the attempts to determine the role of glucose metabolism in tumour growth. As an example Valk *et al.* (1988) have shown a significant difference in the metabolism of glucose in primary cerebral tumours which have been successfully treated and are mostly necrotic in comparison to those which continue to grow apparently unabated. Similar results have been obtained by Haberkorn *et al.* (1990) monitoring radiotherapy of colonic carcinoma. Here patients responding to treatment show a marked reduction in glucose metabolism in metastases. The same group have shown (Knopp *et al.*, 1990) reductions in glucose metabolism of lung tumours responding to radio- or chemotherapy. Alavi *et al.* (1988) have measured a strong correlation between initial glucose metabolism and prognosis for patients with primary cerebral tumours, the result appearing stronger than that predicted by a simple histological grading system.

Amino acid metabolism measurements in glioma also show potential useful results. The high contrast achieved in images due to the low amino acid demand of normal brain has enabled Erikson *et al.* (1987) to show more accurately the growing edge of cerebral tumours using PET images of $C^{11}$-1-Methionine (CMT). It may be that the metabolism of essential amino acids can give a signal related to protein synthesis rates in tumours, making these measurements a sensitive marker of the early effects of therapy at the cellular level. An example of the use of CMT is shown in Bergstrom *et al.* (1987) where the response of prolactinomas to bromocriptine is shown within hours of the start of treatment.

The effects of blood-brain-barrier permeability on drug access have been studied by Ott *et al.* (1990) using PET and $Ga^{68}$-EDTA. This study has shown that there are rapid changes in permeability at the site of primary cerebral lymphomas during chemotherapy. It appears likely that local access of drugs to the tumour is radically impaired 4-5 weeks after treatment implying that drug treatment protocols for cerebral tumours require careful planning.

Received and accepted 5 November 1990.

*Cellular proliferation*

Another area of growing interest in the applications of PET to oncology is the possibility of measuring cell proliferation rates *in situ* using $F^{18}$-fluorodeoxyuridine (FUDR) or $C^{11}$-thymidine (TDR). Wilson *et al.* (1990) have shown a strong correlation between FUDR (an RNA label) uptake in glioma and the histopathology of the tumour. In particular they show that the growing edge of the tumour is well defined by this agent. However the complex *in vivo* metabolism of this agent has made it difficult to establish whether the localisation mechanisms is RNA labelling or due to amino acid metabolites.

Two groups at Seattle, USA and Leuven, Belgium are investigating the use of TDR. The former have shown (Shields *et al.*, 1990) that within 1 h of administration to the dog 40–80% of the TDR is incorporated into DNA. Both groups are presently working on methods of labelling TDR in the ring-2 position as reported by Vander Borght *et al.* (1990) to minimise the signal from metabolites. The images will then give a more accurate measure of DNA incorporation.

*Anti-cancer drug kinetics*

The radiolabelling of anti-cancer drugs will enable measurements of *in vivo* drug kinetics to be made using PET imaging. Dimitrakopoulou *et al.* (1990*a,b*) have imaged tracer dose of $F^{18}$-5FU given to patients with liver metastasis from colonic carcinoma. These data show a strong correlation between the initial uptake of the tracer in the metastases and the response to 5FU therapy. Yang *et al.* (1990) have recently labelled tamoxifen with $F^{18}$ and the group at ICR have labelled iodo-tamoxifen with $I^{124}$ allowing the possibility of pre-therapy tracer studies leading to dosimetry. It is also possible of course to measure the uptake of radiolabelled drugs in normal tissues which will allow a more accurate prediction of toxicity to the kidneys, for instance, to therapy doses. With the growing importance of systemic drug therapy such methods of predicting drug uptake in individual patients are likely to become of increasing value in managing limited resources.

*Receptor ligand imaging*

The study of the role of PET receptor ligands in oncology is still in its infancy, but initial reports indicate some success in both localising tumours with appropriate ligands and in determining receptor density from images. Muhr *et al.* (1986) report the successful monitoring of the effects of bromocriptine treatment on pituitary tumours using $C^{11}$-N-methyl-spiperone to visualise and quantify dopamine-D2 receptor binding. Similar results were also obtained with $C^{11}$-raclopride (Muhr *et al.*, 1987).

Mintun *et al.* (1988) have developed several steroid receptor ligands to quantify oestrogen and progestin receptors in breast cancer. Using $F^{18}$-fluoroestradiol they show that the uptake in primary breast masses as measured from PET images correlates well with tumour oestrogen receptor concentration determined *in vitro*. They have also shown that this tracer successfully localises involved nodes and distant metastases and hence may be of value in assessing the likely sensitivity of these sites to hormone therapy. The use of steroid receptor ligands to monitor hormone therapy via PET images is a real possibility and may be of particular value in patients with widespread disease.

*Tumour targeting*

Tumour targeting with both protein and non-protein based agents is of growing interest because of both the diagnostic and therapeutic potential. PET imaging of such agents may provide improved tumour localisation and dosimetry for systemic therapy. Ott *et al.* (1987) and Flower *et al.* (1989) have shown how imaging of $I^{124}$ in patients with hyperthyroidism and thyroid cancer can give both improved images and allow accurate dosimetry to be carried out. In the former case it has now been possible to produce a dose–response relationship from the *in vivo* studies. Similar results are now becoming available for the latter. This work, has now been extended to the use of $I^{124}$-metaiodobenzylguanadine (mIBG) in patients with neural crest tumours. Preliminary data show that subtherapeutic radiation doses are likely to be achieved in most cases in patients with pheochromocytoma.

An obvious area of interest will be the use of positron emitter labelled monoclonal antibodies to add improved sensitivity and spatial resolution to the purported specificity of these agents to tumours. Bakir *et al.* (1990) report successful *in vitro* and laboratory based studies using $I^{124}$-labelled ICR12, and c-*erb*B2 proto-oncogene product antibody which may prove to be a useful prognostic tool in breast cancer. Wilson *et al.* (1990) have quantitated the uptake of the anti-EMA antibody HMFG1 in both primary and locally recurrent breast tumours again using $I^{124}$ as the tracer label. They show that uptake in these tumours is generally only a little larger than that obtained with a non-specific control and found no correlation between tumour uptake and blood flow as measured using $O^{15}$-water.

A final interesting result comes from Seattle (Koh *et al.*, 1990) who have used the tracer $F^{18}$-fluoromisonidazole in an attempt to determine the hypoxic fraction in tumours. Preliminary studies in dog osteosarcomas have been extended to patients with tumours of the head and neck. Positive uptake of the tracer in excess of blood activity levels have been seen, but the relationship of this result to the binding to hypoxic cells has yet to be ascertained.

**Conclusions**

There are already some areas in which PET may have some direct clinical input into oncology. The differentiation between benign conditions such as radiation induced necrosis and tumour regrowth is of obvious importance in a wide range of tumour sites. Studies have shown that both $F^{18}$-FDG and amino acids are appropriate tracers here. Measurements of protein synthesis or cellular proliferation *in vivo* should provide important methods for determining those patients most likely to benefit from accelerated radiotherapy. Similarly anti-cancer drug dosimetry could well target expensive treatment to responding patients and save non-responders from the worst of the side effects.

Whilst PET is still an expensive technique there will be five centres in operation in the UK in the next 2 years and there should be further possibilities to expand on a broader front (Ott, 1987) by sharing radiopharmaceutical facilities on a regional basis. The unique, quantitative, functional information provided by PET makes it an exciting addition to the imaging tools available to the oncologist to help improve both diagnosis and therapy.

### References

ALAVI, J.B., ALAVI, A., CHAWLUK, J. & 4 others (1988). Positron emission tomography in patients with glioma. A predictor of prognosis. *Cancer*, **62**, 1074.

BAKIR, M.A., BABICH, J.W., STYLES, J.M., DEAN, C.J., ECCLES, S.A. & LAMBRECHT, R.M. (1990). Iodine-124-labelled-ICR12, a new monoclonal antibody for imaging proto-oncogene expression in breast cancer using PET: optimisation of labelling efficiency and immunoreactivity. *J. Nuclear Med.*, **31**, 777.

BEANEY, R.P. & LAMMERTSMA, A.A. (1985). Use of PET in oncology. In *Positron Emission Tomography*, Reivich, M. & Alavi, A. (eds). p 425–450. Liss: New York.

BEANEY, R.P. (1987). Some biological aspects of soft tissue tumours as studied by PET. In *Clinical Efficacy of Positron Emission Tomography*. Heiss, W.-D. (ed.) p. 361–370. Nijhoff: Dordrecht.

BERGSTROM, M., MUHR, C., LUNDBERG, P.O. & 4 others (1987). Rapid decrease in amino acid metabolism in prolactin-secreting pituitary adenomas after bromocriptine treatment. A PET study. *J. Computer Assisted Tomog.*, **11**, 815.

BROOKS, D.J., BEANEY, R.P., LAMMERTSMA, A.A. & 7 others (1986). Glucose transport across the blood brain barrier in normal subjects and patients with cerebral tumours studied using C-11 3-0-Methyl-D-glucose and positron emission tomography. *J. Cerebral Blood Flow & Metabolism*, **6**, 230.

DIMITRAKOPOULOU, A., STRAUSS, L.G., HABERKORN, U. & 5 others (1990*a*). PET evaluation of modulated fluorouracil (FU) chemotherapy in patients with metastatic colorectal carcinoma. *J. Nucl. Med.*, **31**, 803.

DIMITRAKOPOULOU, A., STRAUSS, L.G., HABERKORN, U. & 4 others (1990*b*). PET studies with F-18-uracil in patients with liver metastases from colorectal carcinomas. *J. Nucl. Med.*, **31**, 888.

ERIKSON, K., LILJA, A. & BERGSTROM, M. (1987). Positron emission tomography with C-11-methionine in brain tumours: methionine kinetics, tumour delineation and follow-up studies after therapy. In *Clinical Efficacy of Positron Emission Tomography*. Heiss, W.-D. (ed.) p. 379–390. Nijhoff: Dordrecht.

FLOWER, M.A., SCHLESINGER, T., HINTON, P.J. & 6 others (1989). Radiation dose assessment in radioiodine therapy. 2. Practical implementation using quantitative scanning and PET with initial results on thyroid carcinoma. *Radiotherapy & Oncol.*, **15**, 345.

HABERKORN, U., STRAUSS, L.G., KIMMIG, B. & 4 others (1990). F-18-deoxyglucose imaging with PET in irradiated patients with recurrent colorectal malignancies. *Eur. J. Nuclear Med.*, **16**, S190.

KNOPP, M.V., STRAUSS, L.G., HABERKORN, U. & 6 others (1990). Optimising therapy management in unresectable bronchiogenic carcinoma by metabolic imaging with PET. *J. Nucl. Med.*, **31**, 766.

KOH, W.J., RASEY, J.S., EVANS, M.L., GRIERSON, J.R., KROHN, K.A. & LEWELLAN, T.K. (1990). Imaging of hypoxia in human tumours with (F-18) fluoromisonidazole. *J. Nucl. Med.*, **31**, 756.

MINTUN, M.A., WELCH, M.J., SIEGEL, B.A. & 4 others (1988). Breast Cancer: PET imaging of estrogen receptors. *Radiology*, **169**, 45.

MUHR, C., BERGSTROM, M., LUNDBERG, P.O. & 5 others (1986). Dopamine receptors in pituitary adenomas: PET visualisation with C-11-N-methyl-spiperone. *J. Computer Assisted Tomog.*, **10**, 175.

MUHR, C., BERGSTROM, M., LUNDBERG, P.O., BERGSTROM, K., THUOMAS, K.A. & LANGSTROM, B. (1987). Dopamine receptors in pituitary adenomas and effect of bromocriptine – evaluation with PET and MRI. In *Clinical Efficacy of Positron Emission Tomography*. Heiss, W.-D. (ed.) p. 361–400. Nijhoff: Dordrecht.

OTT, R.J., BATTY, V., WEBB, S. & 8 others (1987). Measurement of radiation dose to the thyroid using positron emission tomography. *Br. J. Radiol.*, **60**, 245.

OTT, R.J., BRADA, M., FLOWER, M.A., BABICH, J.W., CHERRY, S.R. & DEEHAN, B.J. (1990). Measurements of blood-brain-barrier permeability in patients undergoing radiotherapy and chemotherapy for primary cerebral tumours. Submitted to *European Journal of Cancer*.

PATRONAS, N.J., DI CHIRO, G., BROOKS, R.A. & 9 others (1982). Work in progress: [F-18] fluorodeoxyglucose and positron emission tomography in the evaluation of radiation necrosis of the brain. *Radiology*, **144**, 885.

PHELPS, M.E., MAZZIOTTA, J.C. & SCHELBERT, H.R. (1986) (eds). *Positron Emission Tomography and Autoradiography. Principles and Applications to the Brain and Heart.* Raven Press: New York.

SHIELDS, A.F., KOZELL, L.B., LINK, J.M., KOZAWA, S.M. & GARMESTANI, K. (1990). Comparison of PET imaging using (C-11) thymidine labelled in the ring-2 and methyl positions. *J. Nucl. Med.*, **13**, 794.

SNM (1990). *J. Nucl. Med.*, **31**, 707.

VANDER BORGHT, T., LABAR, D., PAUWELS, S., LAMBOTTE, L. & BECKERS, C. (1990). Synthesis of (2-C-11) thymidine; an imaging agent for cellular proliferation. *J. Nucl. Med.*, **31**, 816.

VALK, P.E., BUDINGER, T.F., LEVIN, V.A., SILVER, P., GUTIN, P.H. & DOYLE, W.K. (1988). PET of malignant cerebral tumours after interstitial brachytherapy. *J. Neurosurg.*, **69**, 830.

WILSON, C.B. (1989). Metabolic imaging of human brain tumours. *Seminars in Neurology*, **9**, 388.

WILSON, C.B., SNOOK, D.E., DHOKIA, B. & 7 others (1990). Quantitative measurement of monoclonal antibody distribution and blood flow using Positron Emission Tomography and I-124 in patients with breast cancer. *Internat. J. Cancer* (in press)

YANG, D., EMRAN, A., TANSEY, W. & 6 others (1990). Radiosynthesis of fluorotamoxifen analogues. *J. Nucl. Med.*, **31**, 903.